# Editorial Notes

**Published:** 1941

**Authors:** 


### Editorial Notes

**Mr. H. Edmond G. Boyle.**

The death of Mr. H. Edmond G. Boyle, late Senior Anæsthetist to St. Bartholomew's Hospital reminds us that in 1902 he was Casualty Officer at the Bristol Royal Infirmary. Throughout his professional life he retained a keen interest in the Bristol Royal Infirmary and used to enquire eagerly after affairs in Bristol. Born in Barbados, Boyle felt the old link between Bristol and the West Indies with a strong attachment. His work in the field of anæsthetics won world-wide recognition. He was a cheery companion and stout friend. We offer our sympathy to his widow.

**Bristol Division Meetings.**

The Bristol Division of the British Medical Association has made a successful departure from custom in holding meetings in mid-afternoon during some of the summer months. At each of the two meetings whose proceedings are reported in this issue of the *Journal*, the attendance was over 150. The meetings were open to all medical practitioners, that is to say, to non-members of the Bristol Division and to non-members of the Association. By the courtesy of the University authorities, tea was served in the Physics Department after the meetings. No doubt the subjects set down for discussion and the names of those taking part were the principal attractions: but the time of day and the prospect of getting home long before "black-out" made it possible for doctors to come into Bristol from far distances.

It is evident that whilst meetings in the winter evenings cannot continue, an audience can be found willing and able to attend during the long daylight hours of "double summer time," when black-out is not due until nearly midnight by the clock.

**Medical Privilege and Responsibility.**

In the present war the medical profession has been placed in a privileged position. Whilst the Military Service Act applies to doctors just as to all other citizens, the machinery for deciding which individual members of the profession are to be called upon to do military service has been placed under the management of the medical profession. Through the Central Medical War Committee and the various local committees, "calling-up" of doctors is determined according to the needs of the civil community and the fighting services. Those who are allocated to military service go where they are sent by the

Military Authorities. Up to the present time no compulsion has been applied to those who are left undisturbed in private practice. The Local Emergency Medical War Committee is entrusted with the task of seeing that there are sufficient private practitioners for the needs of each area. The task of this Committee has never been easy and in Bristol there are difficulties which were unforseen. In some districts no doctors reside at nights, and these districts have suffered in the past and may suffer again in the future from intensive bombing. No authority exists to compel doctors to stay during the night in localities where they have surgeries and practise by day. But experience has shown the immense value of having doctors at hand who can attend "incidents" in the vicinity. First aid posts, mobile first aid posts and first aid points cannot be available at every "incident."

Attention was first drawn to this need by the private practitioners of Bristol. They presented a strong case and it was agreed that they were right.

Now the Ministry of Health and all local authorities are urging the desirability of doctors being available to attend where required at "incidents."

But it is essential for the proper organization of such a service of doctors at "incidents" that no large districts should remain without some doctors resident in them at nights. True, no legal compulsion exists, but there would seem to be an honourable duty to be discharged to the community.

The question for the medical profession is how best to discharge this duty.

One solution might be to canvass the profession and call for volunteers to undertake to reside at nights in the doctorless districts, primarily those who have surgeries there, another might be to establish more first aid posts in such districts. In any event, it is desirable that the difficulty should be overcome by voluntary effort rather than by compulsion. No doctor in Bristol would like to see the power enforced which exists under the Defence Regulations, and to receive such an order as the following from the Ministry of Labour and National Service :—

To.............................................................................

In pursuance of Regulation 58A of the Defence (General) Regulations, 1939, I, the undersigned, having been duly authorized in writing by the Minister of Labour and National Service, do hereby on the said Minister's behalf direct you to perform the following service :—

Description of service.............................................................................

(Signed).............................................................................

*National Service Officer.*

Yet the authority to issue these orders already exists.

